# Literature review in the treatment of calciphylaxis: A case with uncontrolled and severe secondary hyperparathyroidism

**Published:** 2016

**Authors:** Ali Abbas Ozdemir, Murat Altay, Aslan Celebi, Osman Mavis

**Affiliations:** 1 Department of Nephrology, Taksim Education and Research Hospital Istanbul, Turkey.; 2 Department of Internal Medicine Taksim Education and Research Hospital Istanbul, Turkey.

**Keywords:** Calciphylaxis, Hyperparathyroidism, Kidney Failure

## Abstract

**Background:**

Calciphylaxis is a serious disorder often observed in dialysis patients and less frequently in chronic renal failure patients with secondary hyperparathyroidism. Mortality rate increases following the development of calciphylaxis, immediate application of parathyroidectomy along with other treatment options may be lifesaving.

**Case Presentation:**

A 44-year-old male patient had been on regular hemodialysis three times per week, with 4-hour sessions since December 2003. The etiology of his renal failure was unknown and the patient had no systemic disease when hemodialysis was started, painful, erythematous skin lesions were detected over and around the external malleolus of the right foot. In the next two months, erythematous skin lesions gained a necrosed character and spread into the malleolar and posterior tibial region and back of the ankle in both extremities. The patient showed no clinical signs of recovery and despite anti-biotherapy, debridement and protective measures, the skin lesions got infected and presented as intolerable, painful wounds. The patient was diagnosed with calcific uremic arteriolopathy (CUA) and hospitalized for parathyroidectomy. All parathyroid glands were removed after having checked quick PTH intraoperatively. A small amount of parathyroid tissue was intramuscularly auto-implanted into the right forearm .Skin lesions showed fast improvement in further follow-ups. Three months after parathyroidectomy, iPTH value was measured as 1197 pg/ml. After 6 months of medical treatment, iPTH was found as 970 ng/L and we decided to remove the implanted parathyroid tissue from the right forearm.

**Conclusion:**

In this article, we present a case of calciphylaxis accompanied by severe secondary hyperparathyroidism.

Calciphylaxis or calcific uremic arteriolopathy (CUA) is a serious disorder often observed in dialysis patients and less frequently in chronic renal failure patients with secondary hyperparathyroidism (1). In this article, we present a case of calciphylaxis accompanying severe secondary hyperparathyroidism. 

## Case presentation

A 44-year-old male patient had been on regular hemodialysis three times per week, with 4-hour sessions since December 2003. The etiology of his renal failure was unknown and the patient had no systemic disease when hemodialysis was started. 

Anamnesis revealed no family history of a serious disease 4 year before coronary bypass surgery for coronary artery disease was performed. The initial iPTH value of the patient measured at the beginning of hemodialysis was 74.5 ng/L (table 1) and his clinical and laboratory findings for renal osteodystrophy were within acceptable limits. His follow-up parameters showed progressively increasing values of iPTH, along with Ca and P. In our patients, kidney transplantation is a good choice. But in our country, a large part of it is made via a living donor. Our patient does not have a living donor and cadaver donor cannot be found. Following phosphate control, calcitriol, paricalcitol and cinacalcet treatment have started. The level of iPTH in start of the cinacalcet in 2010 was 2121. Cinacalcet was first administered at 30 mcg/day in April 2010 for one month, at 60 mcg/day for another month, and when PTH values did not decrease sufficiently at 90 mcg/day for one year unit such time parathyroidectomy occurred. 

**Table I:** Follow-up of biochemical parameters of the patient

**Date**	**PTH (pg/ml)**	**Ca (mg/dl)**	**P (mg/dl)**	**ALP (unit/L)**	**Albumin (g/dl)**
11.04.2003	74.50				
04.12.2003	592.60				
27.01.2005	439				
05.01.2010	1431	9.2	6.8	377	3.9
06.04.2010	2121	8.7	4.5	465	3.8
06.07.2010	1557	9.1	5.3	600	4.1
09.10.2010	2000	10	6.2	277	3.8
04.01.2011	2055	8.3	6.2	868	4
05.04.2011	1654	7.6	5.8	1005	3.9
02.07.2011	2171	7.3	6.3	606	3.7
03.10.2011	1582	9.5	10.1	540	3.9
02.01.2012	2050	9.4	7	1012	3.9
13 days after total parathyroidectomy and right forearm auto-implantation					
26.04.2012	396.60				
02.07.2012	1197	7.6	7.9	181	4
01.10.2012	970	8.4	6.7	211	4.3
03.12.2012	662	8.1	6.4		4.8
After excision of the auto-implanted parathyroid tissue					
02.01.2013	430	7.8	6.1	149	4.6

**Figure 1:**
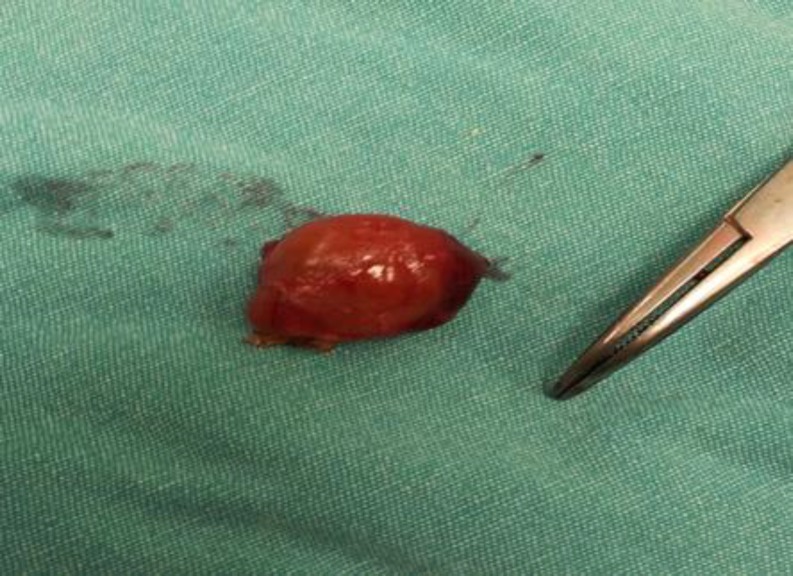
Parathyroid glands after surgery

CaxP values of the patient were consistently above 72 and the patient clinically suffered severe and widespread musculoskeletal pain during this period. Parathyroid MRI was performed when iPTH values went above 2000 ng/L, parathyroid glands were found to be significantly hyperplastic; however, parathyroid scintigraphy was normal. Lesions were 18x21 mm, 14x10 mm and 24x16 mm in size. In April 2011, painful, erythematous skin lesions were detected over and around the external malleolus of the right foot. In the next two months, erythematous skin lesions gained a necrosed character and spread into the malleolar and posterior tibial region and back of the ankle in both extremities. In the lower extremity, Doppler USG the arteries were found to be open and the patient received hyperbaric oxygen therapy for 30 sessions via the recommendation of the dermatology department. The patient showed no clinical signs of recovery and despite anti-biotherapy, debridement and protective measures, the skin lesions got infected and presented as intolerable, painful wounds. The patient was diagnosed with CUA and hospitalized for parathyroidectomy. All parathyroid glands were removed after having checked quick PTH intraoperatively (fig 1). 

**Figure 2:**
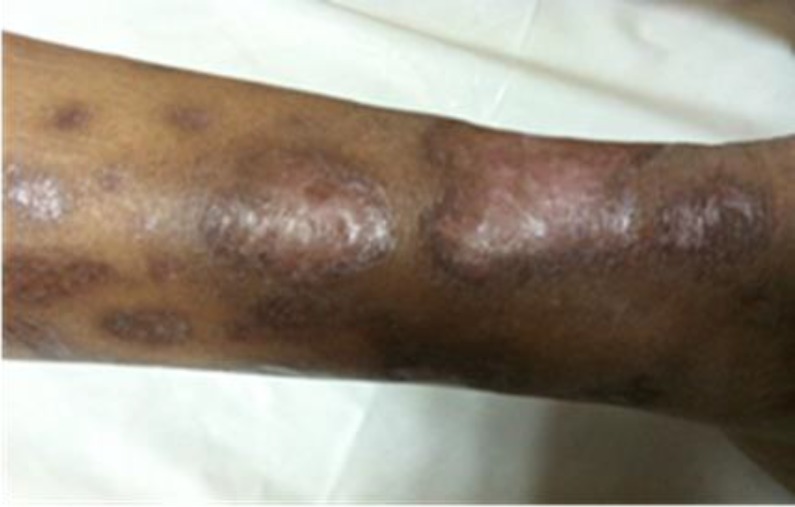
. Skin lesions of Patients

 A small amount of parathyroid tissue was intramuscularly auto-implanted into the right forearm when intraoperative value of the PTH was <300 ng/L. Skin lesions showed fast improvement in further follow-ups (fig 2). On the 13^th^ postoperative day, the iPTH value was 396.60 ng/L. Three months after parathyroidectomy, iPTH value was measured as 1197 pg/ml. After 6 months of medical treatment, iPTH was found as 970 ng/L and we decided to remove the implanted parathyroid tissue from the right forearm. Following surgical excision, the mean iPTH value was measured 430 ng/L.

## Discussion

However, the pathogenesis of CUA has yet to be fully clarified. The mean incidence rate is reported as 1 to 4 per 100 patient years. Female sex, obesity, diabetes, use of warfarin, low albumin levels, calcium-based phosphate binders, vitamin D analogs, and long-term use of high-dose iron salts are the known risk factors (2).

 CUA usually sets off following a local skin trauma or hypotensive attack. Characteristically, it emerges in the dermis, subcutaneous adipose tissue, and less often in the muscle layer as fields of necrosis. Most clinicians diagnose CUA only by physical examination. If clinical picture of the patient is not strongly indicating CUA, lesion biopsy may be performed for diagnosis or differential diagnosis. Peripheral vascular disease, pyoderma gangrenosum, atheroemboli, cryoglobulinemia, warfarin-related skin lesions and systemic oxalosis are conditions in differential diagnosis. 

Hypoalbuminemia, a strong risk factor in emergence of CUA (3), was not detected in the follow-ups of our patient. This might be due to his eating habits and inadaptability to the diet. The tendency of the alkaline phosphatase level increases in line with PTH level indirectly pointing out to the presence of high-turnover bone disease.

Literature mostly includes patients who were medically treated. Despoina et al. successfully treated CUA in a 57-year-old woman, using cinacalcet and paricalcitol systemically and autologous growth factors locally (4). Although we did not observe a significant decrease in PTH levels during the early stages of our cinacalcet treatment in our case, PTH level started to decline significantly by gradually increasing doses later on. However, despite taking cinacalcet and other conventional treatments, PTH level did not fall below 1440. The patient was then given sodium thiosulfate and finally treated with hyperbaric oxygen therapy. A parathyroidectomy was not necessary. In another study, total parathyroidectomy was performed after having checked intraoperative rapid PTH levels and auto-implantation on 22 patients whose PTH levels were critically low. Hyperparathyroidism findings were not detected in almost any patient in their long-term follow-ups (5). Similarly, after checking intraoperative rapid PTH levels, we performed auto-transplantation on our case and the reimplanted tissue was excised under local anesthesia when PTH level went above 1000.

 The occurrence of CUA in our case, who has been receiving hemodialysis treatment for about 10 years, should not be unexpected considering the PTH levels and Ca-P statuses through these years. High levels of phosphorus since the beginning, despite the treatment, may be related to weariness due to constant dieting, similar to that in patients who have been receiving dialysis treatment for years. In our opinion, persistent PTH level above 1000, despite combined treatment of cinacalcet and other therapies, is an important risk factor for the development of CUA. Near-total parathyroidectomy or total parathyroidectomy with reimplantation of some parathyroid tissue in the forearm before calciphylaxis develop might be a rational treatment method. 

As the mortality rate increases following the development of calciphylaxis, immediate application of parathyroidectomy along with other treatment options may be lifesaving. As it can be clearly seen in the literature, further prospective controlled studies are needed.

**Implications for Practice:**

Because of the nature of disease, CUA is a fatal disease; carries high mortality and morbidity. The mortality rate of calciphylaxis is about 60-80% (6). In conclusion, conventional treatment stages and complications associated with the SHP were followed sequentially. When the patients referred to us, calciphylaxis was developed and we suggest parathyroidectomy in those with plasma calcium and phosphate concentrations which are uncontrollable with medical therapy. As the mortality rate increases following the development of calciphylaxis, immediate application of parathyroidectomy along with other treatment options may be lifesaving.
